# Subtle Alterations in Brain Anatomy May Change an Individual’s Personality in Chronic Pain

**DOI:** 10.1371/journal.pone.0109664

**Published:** 2014-10-07

**Authors:** Sylvia M. Gustin, Jamie G. McKay, Esben T. Petersen, Chris C. Peck, Greg M. Murray, Luke A. Henderson

**Affiliations:** 1 Neuroscience Research Australia, Randwick, NSW, Australia; 2 Department of Anatomy and Histology, University of Sydney, Sydney, NSW, Australia; 3 Departments of Radiology and Radiotherapy, University Medical Center Utrecht, Utrecht, The Netherlands; 4 Faculty of Dentistry, University of Sydney, Sydney, NSW, Australia; University of Texas at Dallas, United States of America

## Abstract

It is well established that gross prefrontal cortex damage can affect an individual’s personality. It is also possible that subtle prefrontal cortex changes associated with conditions such as chronic pain, and not detectable until recent advances in human brain imaging, may also result in subtle changes in an individual’s personality. In an animal model of chronic neuropathic pain, subtle prefrontal cortex changes including altered basal dendritic length, resulted in altered decision making ability. Using multiple magnetic resonance imaging techniques, we found in humans, although gray matter volume and on-going activity were unaltered, chronic neuropathic pain was associated with reduced free and bound proton movement, indicators of subtle anatomical changes, in the medial prefrontal cortex, anterior cingulate cortex and mediodorsal thalamus. Furthermore, proton spectroscopy revealed an increase in neural integrity in the medial prefrontal cortex in neuropathic pain patients, the degree of which was significantly correlated to the personality temperament of novelty seeking. These data reveal that even subtle changes in prefrontal cortex anatomy may result in a significant change in an individual’s personality.

## Introduction

It is well established that gross prefrontal cortex damage can alter an individual’s personality. Less recognized is the possibility that in individuals with conditions such as chronic pain, subtle changes in prefrontal cortex anatomy associated with the condition, may also result in subtle changes in an individual’s personality. The lack of recognition of a connection between personality and brain anatomy, likely stems from the fact that until recently, it was almost impossible to assess subtle changes in brain anatomy in living humans. The recent use of more sensitive brain imaging techniques has revealed that chronic pain is associated with subtle anatomical alterations in the thalamus, insular and cingulate cortices [1], [2], [3]. It is also evident that chronic pain is associated with subtle, less well known, cognitive changes. For example, subjects with chronic pain display impaired emotional-based decision making [4], [5], that is, they have decreased ability to think clearly and make advantageous decisions. In addition, it has recently been reported that chronic pain subjects display altered personality temperaments such as increased harm avoidance, i.e. excessive worrying, and low novelty seeking, i.e. reduced appetite for new experiences and reduced impulsive decision making [6].

It has been proposed that cognitive changes associated with chronic pain may result from alterations in activity within cortical areas such as the prefrontal cortex [7], [8], [9], [10]. In a recent animal investigation, Metz and colleagues [11] found that in the spared nerve injury model of neuropathic pain, basal dendrites of pyramidal neurons within the mPFC were longer and had more branches than in sham-operated rodents. In addition, pain was associated with increased spine density and increased contribution of the NMDA component of synaptic currents. If similar changes in mPFC anatomy occur in humans, they may underlie at least in part the altered temperament changes such as reduced novelty seeking and increased harm avoidance that occur in human subjects with chronic pain.

The study aims to explore anatomical and biochemical changes within the mPFC and related brain regions in individuals with chronic pain and to determine if any changes are associated with an individual’s personality. Diffusion tensor imaging (DTI) and T2-relaxometry was used to detect subtle changes that indicate alterations in the physical organization and myelination and/or development of axons and dendrites [12], [13]. Additionally, spectroscopy can reveal biochemical changes, e.g. N-acetyl aspartate (NAA), an indicator of neuronal viability [14]. We hypothesize that in chronic pain subjects, the mPFC will display decreased DTI and T2 relaxation parameters and increased NAA levels and that these changes will be significantly related to an individual’s personality temperament of novelty seeking and harm avoidance. Such alterations would show that this region has undergone subtle changes in neuronal anatomy consistent with increased dendritic spine lengths, dendritic spine numbers and neuronal cell bodies.

## Methods

Twenty two subjects with chronic pain (painful trigeminal neuropathy; 18 females, mean age 49.8±1.8 years [±SEM]) and 43 healthy controls without ongoing pain (35 females; mean age 46.4±2.7 years) were recruited at the University of Sydney, Australia. All chronic pain subjects were diagnosed with trigeminal neuropathy according to the Liverpool criteria [15]. After complete description of the study to the subjects, written informed consent was obtained for all procedures and the study was approved by Institutional Human Research Ethics Committees, University of Sydney. Some of the chronic pain subjects used in this study were also used in previous investigations [1], [16], [17], [18], [19].

### Psychophysical Measures

Each chronic pain patient rated their on-going pain intensity with a vertical pencil stroke on a 10 cm horizontal line (visual analogue scale [VAS]; 0 cm = “no pain” to 10 cm = “maximum imaginable pain”) three times a day for the week prior to the scanning session. These pain values were averaged to provide an indication of each subject’s chronic pain rating. Each chronic pain subject also completed a McGill Pain Questionnaire and drew a distribution map of their on-going pain.

To assess individual subject’s temperament, 19 chronic pain (16 females; mean age [±SEM]: 53±1.8) and 30 control subjects (24 females; age: 51±1.0) completed the revised version of the temperament and character inventory (TCI-R) [20]. Significant differences between these temperament measures in chronic pain subjects and controls were determined using t tests (p<0.05).

### MRI Acquisition

In all subjects, three 3D T1-weighted anatomical image sets (echo time = 2.5 ms, repetition time [TR] = 5600 ms, flip angle = 8°, voxel size = 0.8×0.8×0.8 mm) and four high-resolution diffusion tensor image sets (TR = 8788ms; flip angle = 90°, voxel size = 2×2×2.5 mm, 32 directions, b = 0 and 1000 s/mm^2^) covering the entire brain were collected using a 3T Philips Intera machine. Three T1-weighted images and 4 DTI series were collected separately for subsequent averaging. In 39 of the 43 control and 19 of the 22 chronic pain subjects, proton density and T2-weighted images covering the entire brain were also collected (TR = 4330 ms; echo times: 20, 40, 60, 80, 100 ms; voxel size = 2×2×2.5 mm). Multiple echo times were used to create multiple images for the subsequent calculation of T2-relaxation maps. In 24 controls and 18 chronic pain subjects, a quantitative arterial spin labelling (QASL) series, encompassing the entire brain was collected (TR/TE/DTI/TI1 = 4000/23/300/40 ms, 64×64 matrix, 14 slices, FOV = 240×240, flip-angle = 35/11.7°, SENSE = 2.5, V_enc_ = [∞, 4 cm/s], 82 (48 @ V_enc_ = 4 cm/s, 24 @ V_enc_ = ∞, 10 low flip angle, all implemented in a two separate sequences) [21]. In addition, a series of T1-weighted anatomical images, at the same slice locations as the QASL images were collected.

All neuropathic pain and healthy subjects were asked to return for a second scanning session. Twelve control (11 females; mean age±SEM: 53.4±3.1) and 11 neuropathic pain subjects (10 females; mean age±SEM: 53.5±2.8) returned for the second scanning session, the remaining subjects declined to return. Proton magnetic resonance spectroscopy (1H-MRS) was performed on the contralateral (to pain) mPFC and on the right mPFC in control subjects (TR = 2000 ms, echo time = 29 ms, 1024 acquisition points, spectral width of 2 kHz, total acquisition time = 9 min, voxel size 20×15×30 mm). The location of the region selected was based on the diffusion and T2 relaxometry results. Automatic shimming (pencilbeam auto first order option) was performed resulting in line widths of <10 Hz for all spectra. There were no significant differences in age (p>0.05, t-test) or gender composition (p>0.05; Chi2 test) in any of the MRI analyses.

### MRI Analysis

#### DTI

All images were processed using SPM8 [22] and custom software. The diffusion-weighted images were motion corrected, coregistered to one another, averaged and diffusion tensors calculated [12]. Mean diffusivity maps were derived, spatially normalized and smoothed (6mm full-width-at-half-maximum [FWHM] Gaussian filter). Significant differences in mean diffusivity between controls and chronic pain subjects were determined (p<0.05, random effects, family wise error corrected for multiple comparisons, minimum cluster 30 voxels, age and gender nuisance variables). Mean diffusivity values of significantly different clusters were extracted and significance verified (2 sample t-tests, p<0.05). Significant correlations between mean diffusivity and pain intensity, pain duration, and temperament values (novelty seeking etc) were determined (p<0.05). Significant differences in r-values in chronic pain compared with controls were also determined using Fisher r-to-z transformation (p<0.05).

#### T2 relaxometry

The following equation was used to calculate T2 brain maps: T_2_ = (TE_2_–TE_1_)/ln(SI_1_/SI_2_) where TE_1_ and TE_2_ are the echo times for proton density and T2-weighted images, and SI_1_, SI_2_ represent proton density and T2-weighted image signal intensities, respectively. A ceiling threshold of 500 ms was applied to eliminate cerebrospinal fluid. In each subject, one of the T2-weighted images (TE = 60 ms) was spatially normalized and the parameters applied to the T2 map. The spatially normalized T2 map was smoothed (6 mm FWHM) and significant differences in T2 relaxation times between controls and chronic pain subjects determined (p<0.05, random effects, family wise error corrected for multiple comparisons, minimum cluster 30 voxels, age and gender nuisance variables). T2 relaxation times of significantly different clusters were extracted and significance verified (2 sample t-tests; p<0.05). Significant correlations between T2 relaxation times and pain intensity, pain duration, and temperament values (novelty seeking etc) were determined (p<0.05). Significant differences in r-values in chronic pain compared with controls were determined using Fisher r-to-z transformation (p<0.05).

#### Voxel based morphometry

T1-weighted anatomical image sets were coregistered to one another, averaged, modulated, spatially normalized, segmented and smoothed (6 mm FWHM). Gray matter volumes from significant mean diffusivity and T2 relaxation clusters were extracted and compared between control and chronic pain subjects (2-tailed, 2-sample t-tests, p<0.05).

#### Quantitative arterial spin labelling

QASL images were opened [21] and cerebral blood flow (CBF) maps created. In addition, anatomical (gray/white) image sets were created from the CBF maps. These anatomical images were then co-registered to the T1-weighted anatomical image set collected at the same slice locations and the resulting parameters applied to the CBF maps. The T1-weighted anatomical images were then normalized and the parameters applied to CBF maps. CBF values from significant mean diffusivity and T2 relaxation clusters were extracted and compared between control and chronic pain subjects (2- tailed, 2-sample t-tests, p<0.05).

#### Proton magnetic resonance spectra (1H-MRS)

MRS data were analyzed in the time domain using the Java-based magnetic resonance user’s interface (jMRUI 4.1, European Union project). The dominant water resonance was removed using the Hankel Lanczos Singular Valve Decomposition algorithm. All metabolite resonances were quantified using QUEST (containing a 29 ms TE metabolite basis set including N-acetylaspartate (NAA), Creatine [23], Aspartate, Glutamate, Glutamine, Myo-Inositol (MI) and Glycerolphosphocholine). Ratios were calculated for NAA relative to Cr and MI relative to Cr. Significant differences in metabolite ratios between chronic pain subjects and healthy controls were determined (t-tests, p<0.05). One-tailed Pearson correlation test was used to determine significant (p<0.05) correlations between NAA/Cr ratios, MI/Cr ratios and pain intensity and pain duration.

## Results

### Pain and Temperament

Chronic pain subjects had on-going orofacial pain with mean pain intensity of 4.0±0.4 and mean pain duration of 5.7±0.9 years (Table 1). Chronic pain subjects had significantly lower mean novelty seeking values ([mean±SEM] controls: 102±2; chronic pain: 95±2) and significantly higher harm avoidance values (control: 89±3; chronic pain: 105±4) compared with controls. In contrast, there were no significant differences in temperament scores of reward dependence (control: 106±2; chronic pain), or persistence (control: 124±4; chronic pain: 117±5).

**Table 1:** Characteristics of chronic pain subjects.

Subject	Age	Gender	Duration of pain (years)	Site	Ongoing pain (10 cm VAS)	Analgesic Medication
1	51	M	3.5	right	2.1	Amitriptyline Hydrochloride
2	48	F	9.0	bilateral	2.5	Gabapentin
3	42	F	2.0	right	5.5	Neurontin
4	64	F	11.0	right	5.0	Gabapentin
5	52	F	3.5	right	4.5	none
6	47	F	5.0	left	1.1	none
7	53	F	2.5	right	1.5	none
8	52	F	1.5	bilateral	6.9	Gabapentin
						Oxycodone
						Paracetamol
9	55	F	2.0	left	5.2	Amitriptyline Hydrochloride
						Gabapentin
						Oxycodone Hydrochloride
						Paracetamol
10	46	M	9.0	bilateral	3.1	none
11	46	F	3.0	bilateral	6.5	Diazepam
						Paracetamol
						Ibuprofen (PRN)
12	42	F	11.0	bilateral	4.8	Carbamazepine
						Paracetamol
13	48	F	1.3	bilateral	2.6	Gabapentin
14	34	M	5.0	bilateral	3.5	Pregabalin Nortriptyline
15	59	F	5.0	left	3.3	Paracetamol
						Ibuprofen
16	54	F	2.0	right	1.6	Amitriptyline
17	44	F	6.5	bilateral	6.4	none
18	40	F	3.5	bilateral	4.0	Carbamazepine
19	67	F	14.0	left	8.4	none
20	43	M	16.0	left	5.8	Amitriptyline
21	44	F	7.0	bilateral	1.2	none
22	65	F	1.5	bilateral	2.5	none
**Mean (±SEM)**	49.8 (±1.8)		5.7 (±0.9)		4.0 (±0.4)	

PRN: *Pro re nata* – “as needed”.

### T2 relaxation times and mean diffusivity

**Figure 1:**
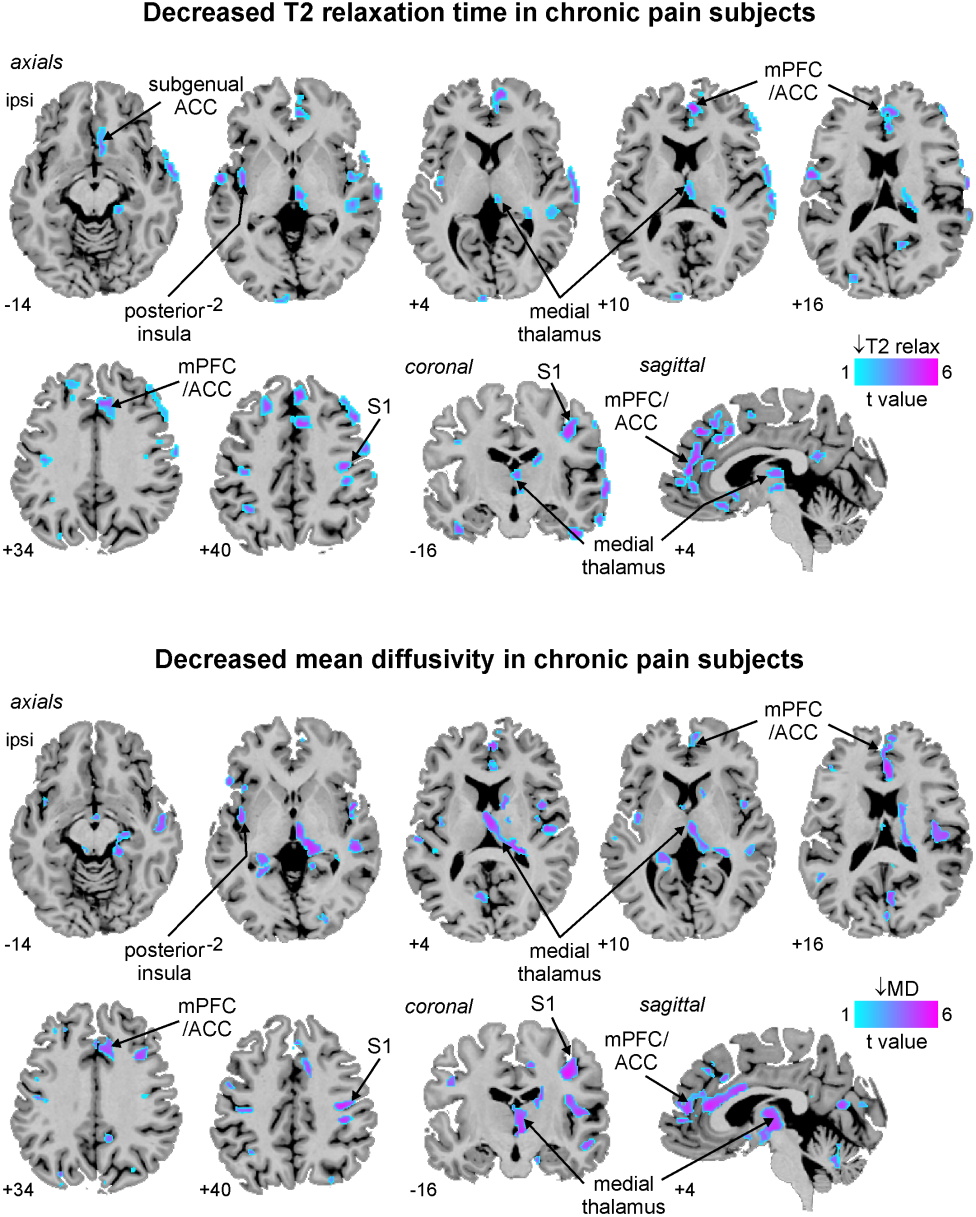
Brain regions in which T2-relaxation times (upper panel) or mean diffusivity values (lower panel) were significantly reduced in subjects with painful trigeminal neuropathy compared with pain-free, healthy controls. Significant reductions are colour-coded according to t-value and overlaid onto axial, coronal and sagittal sections of an individual subject’s T1-weighted image set. Slice locations in Montreal Neurological Institute space are indicated at the lower left of each image. ACC: anterior cingulate cortex; ispi: ipsilateral to side of on-going pain; MD: mean diffusivity; mPFC: medial prefrontal cortex; S1: primary somatosensory cortex.

**Figure 2:**
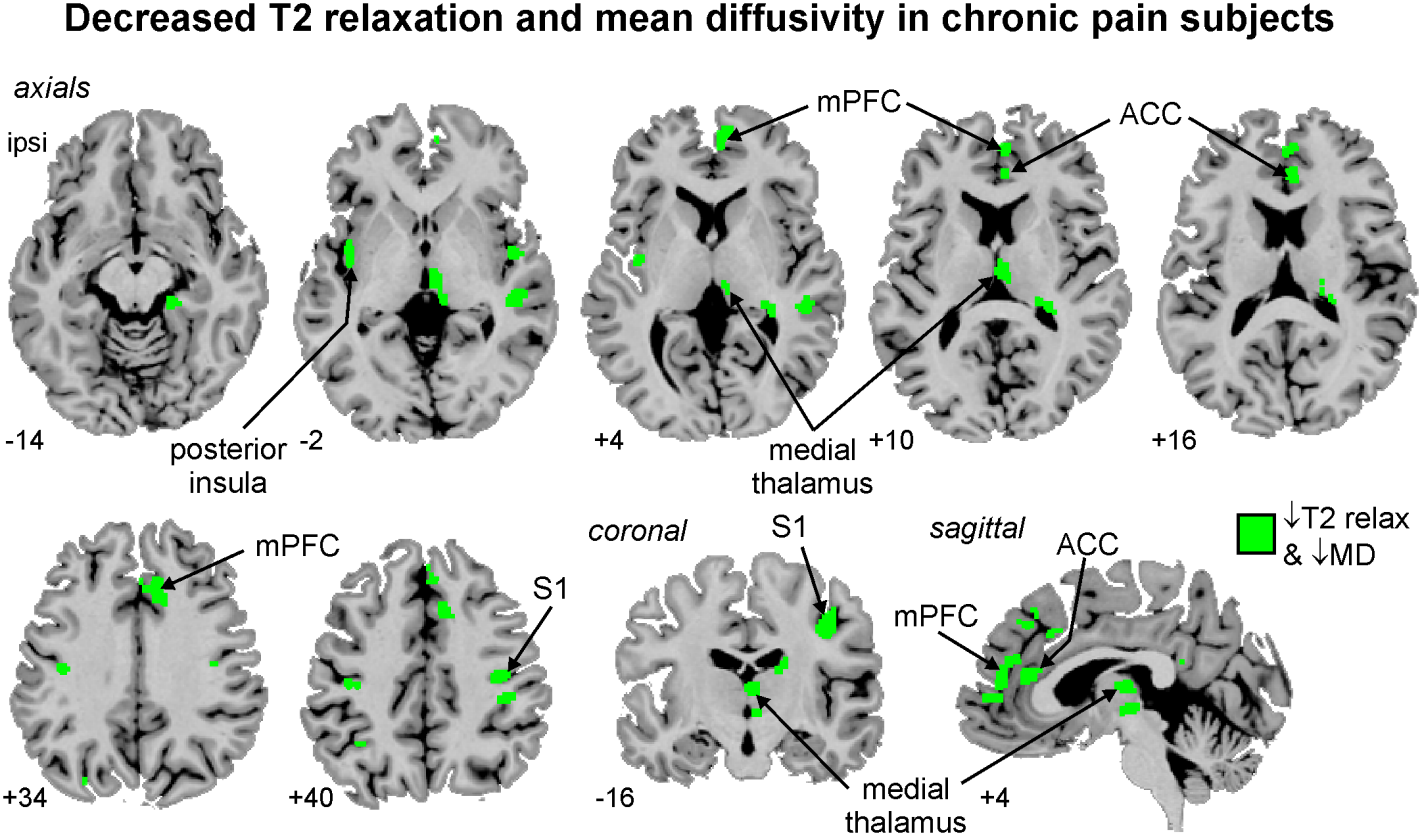
Brain regions in which both T2-relaxation times and mean diffusivity values (MD) were significantly reduced in subjects with painful trigeminal neuropathy compared with pain-free, healthy controls. Areas of significant reductions are shaded green and overlaid onto axial, coronal and sagittal sections of an individual subject’s T1-weighted image set. Slice locations in Montreal Neurological Institute space are indicated at the lower left of each image. ACC: anterior cingulate cortex; ispi: ipsilateral to side of on-going pain; mPFC: medial prefrontal cortex; S1: primary somatosensory cortex.

**Figure 3:**
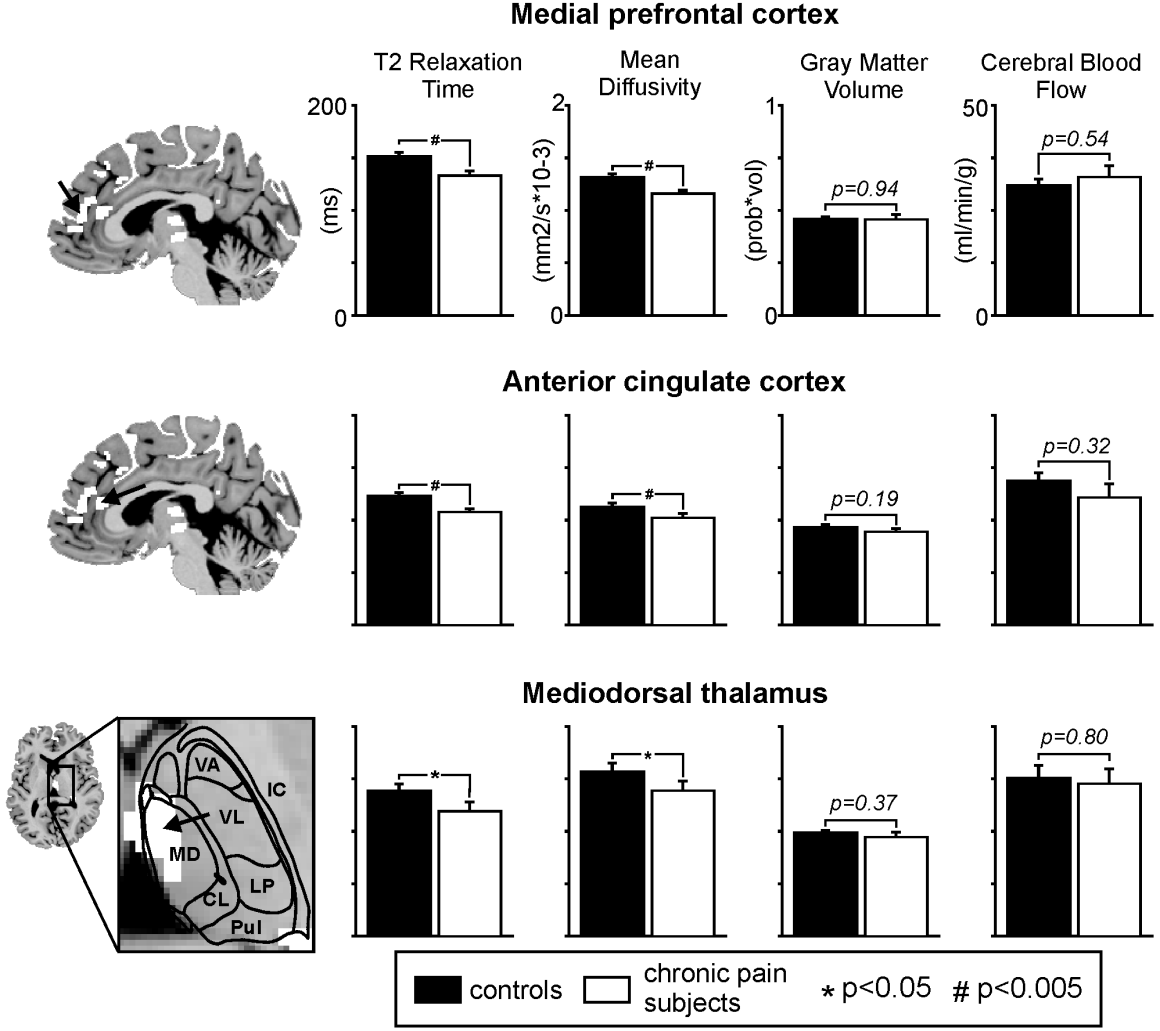
Plots of T2 relaxation times, mean diffusivity, gray matter volume and cerebral blood flow in three brain regions: the medial prefrontal cortex, anterior cingulate cortex and mediodorsal thalamus. Control subjects are shaded black and chronic pain subjects are shaded white. Note that although T2 relaxation times and mean diffusivity is reduced in all three regions, there are no significant changes in either gray matter volume or resting cerebral blood flow. Location of reductions in T2 relaxation times and mean diffusivity are shown to the left and indicated by an arrow. Note the thalamic reductions are located within the mediodorsal nucleus (MD). CL: centrolateral; IC: internal capsule; LP: lateral posterior; Pul: pulvinar; VA: ventral anterior; VL: ventral lateral.

Chronic pain subjects had reduced T2 relaxation times in the contralateral (to pain) subgenual anterior cingulate cortex (ACC), contralateral ACC and medial prefrontal cortices, ipsilateral posterior insula, contralateral medial thalamus and primary somatosensory cortex bilaterally (Figure 1, Table 2). Remarkably, whole brain analysis of mean diffusivity changes revealed significant decreases in these same regions apart from the subgenual ACC. Chronic pain subjects displayed significantly reduced mean diffusivity in the contralateral ACC, mPFC, ipsilateral posterior insula, contralateral medial thalamus (in the region of the mediodorsal nucleus) and primary somatosensory cortex bilaterally (Figure 1, Table 2). Brain regions where chronic pain subjects showed significant decreases in both T2 relaxation times and mean diffusivity are shown in Figure 2. Extraction of T2 relaxation times and mean diffusivity values from regions further confirmed these significant differences (Figure 3). Given the potential influence of medication we compared T2 relaxation times and mean diffusivity values within the mPFC, ACC and thalamus and found no significant differences between those chronic pain subjects taking medication (n = 8) to those who were not (n = 14). Furthermore, given the well-documented connections and functional interactions between the mediodorsal thalamus, mPFC and ACC [24], we restricted most of the remaining analysis on these three brain regions.

**Table 2:** T2 relaxation, mean diffusivity, grey matter volume and cerebral blood flow values within the contralateral medial prefrontal cortex (mPFC), anterior cingulate cortex (ACC), mediodorsal thalamus, ipsilateral posterior insula and contralateral primary somatosensory cortex (S1).

		contralateralmPFC	contralateralACC	contralateralmediodorsalthalamus	ipsilateralposteriorinsula	contralateral S1
**T2 relaxation**	controls	151±4	125±2	143±6	149±4	126±4
**time (ms±SEM)**	chronicpain	132±4*	110±3*	118±7*	124±5*	104±4*
**Mean diffusivity**	controls	1.31±0.03	1.15±0.02	1.56±0.05	1.28±0.06	1.16±0.06
**(mm^2^/sx10^−3^±SEM)**	chronicpain	1.16±0.03*	1.02±0.02*	1.36±0.04*	1.14±0.04	1.02±0.02*
**Grey matter volume**	controls	0.46±0.01	0.47±0.01	0.49±0.01	0.64±0.01	0.36±0.01
**(prob** * **vol ±SEM)**	chronicpain	0.46±0.01	0.45±0.01	0.48±0.02	0.64±0.02	0.38±0.01
**Cerebral blood flow**	controls	31.1±1.8	34.5±2.2	37.9±3.1	29.2±2.3	38.6±2.8
**(ml/min/g ±SEM)**	chronicpain	33.0±2.6	30.2±3.6	36.7±3.6	28.8±3.3	34.9±3.3

*significantly different to control subjects (p<0.05).

### Gray matter volumes and baseline blood flow

Voxel based morphometry of T1-weighted anatomical images revealed that chronic pain was not associated with significant gray matter volume changes within the mPFC, ACC, mediodorsal thalamus, dorsal insula, or primary somatosensory cortex. Similarly, assessment of baseline regional cerebral blood flow using QASL revealed no significant difference in cerebral blood flow in chronic pain subjects compared with controls in the mPFC, ACC, mediodorsal thalamus, dorsal insula or primary somatosensory cortex (Figure 3, Table 2).

### Proton magnetic resonance spectra (1H-MRS)

**Figure 4:**
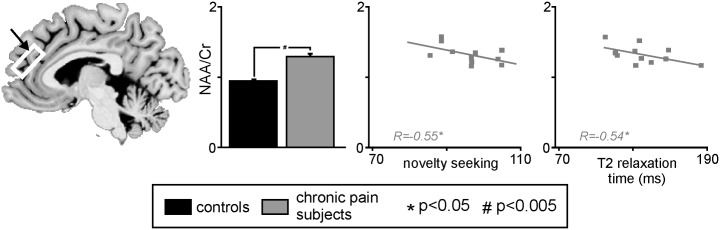
Sagittal slice showing location from which proton spectroscopy was performed in the contralateral medial prefrontal cortex (mPFC) of subjects with chronic pain and pain-free controls. The bar graph shows mean (±SEM) NAA/Cr values for chronic pain patients and healthy controls. To the right are two plots of novelty seeking against mPFC NAA/Cr and mPFC T2 relaxation times against mPFC NAA/Cr. Note that both novelty seeking and mPFC T2 relaxation times are significantly correlated to NAA/Cr within the mPFC. NAA: N-acetyl aspartate; Cr: creatine.

Finally, to assess biochemical changes within the mPFC, proton spectroscopy was performed. Within the contralateral mPFC, spectral analysis revealed that chronic pain subjects had significant elevated levels of N-acetyl aspartate/Creatine (NAA/Cr) compared to controls (mean±SEM NAA/Cr: controls: 1.16±0.03, chronic pain: 1.32±0.04; p<0.005) (Figure 4). No significant difference in myo-inositol/Cr (MI/Cr) was found in the mPFC of chronic pain subjects compared to controls (mean±SEM MI/Cr; controls: 0.78±0.03, chronic pain: 0.72±0.02; p>0.05).

### Relationships with pain and temperament

**Figure 5:**
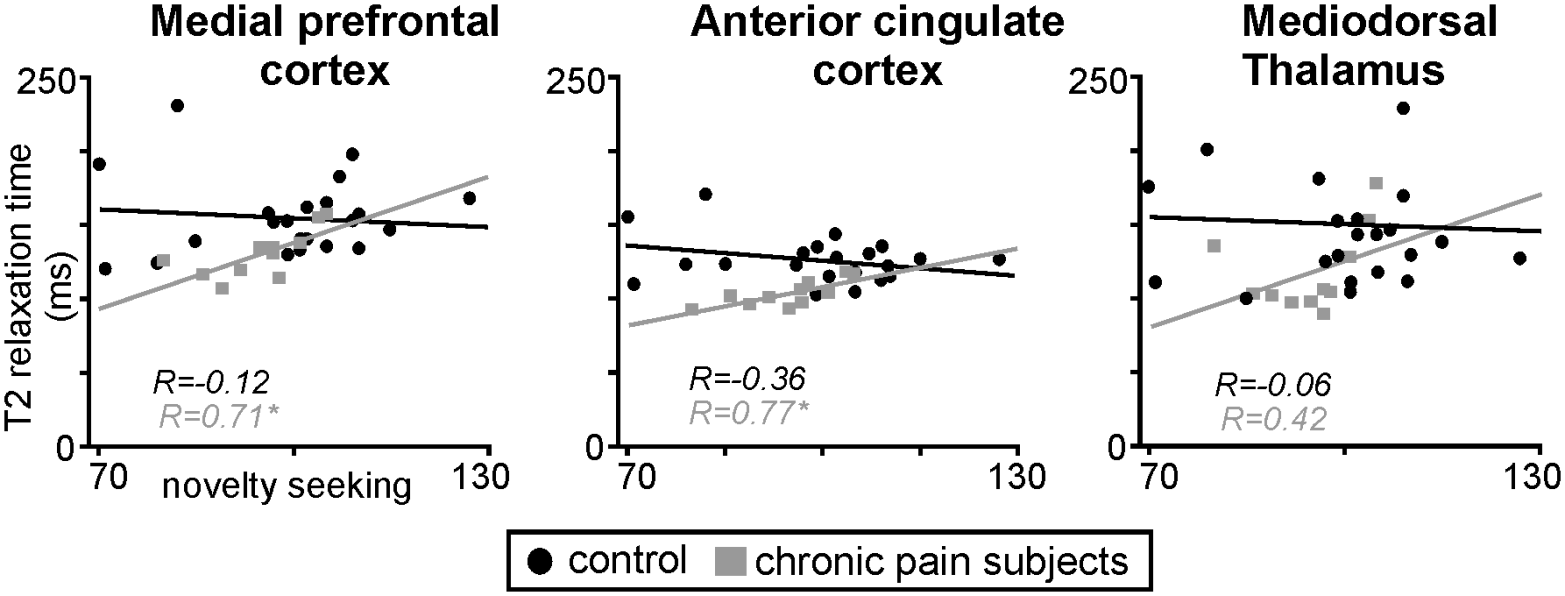
Plots of T2 relaxation times against novelty seeking temperament in control subjects (black circles) and in chronic pain subjects (gray squares) within the medial prefrontal cortex, anterior cingulate cortex and mediodorsal thalamus. Note that in chronic pain subjects, T2 relaxation times in the medial prefrontal and anterior cingulate cortices are significantly negatively correlated to novelty seeking. No such relationship was found in controls. *indicates significant difference in r values between controls and chronic pain subjects determined using Fisher r-to-z transformation.

Within the mPFC, ACC and thalamus, T2 relaxation times and mean diffusivity values in chronic pain subjects were not significantly correlated with either on-going pain intensity or pain duration. In contrast, within the mPFC and ACC, T2 relaxation times were positively correlated to novelty seeking (mPFC: r = 0.71; ACC: r = 0.77) (Figure 5). That is, the greater the decrease in T2 relaxation times, the lower the individual’s novelty seeking score. In contrast, no significant relationship occurred between T2 relaxation times and novelty seeking within the thalamus (r = 0.42), or between T2 relaxation times and harm avoidance in all three regions (mPFC: r = −0.12; ACC: r = −0.36; mediodorsal thalamus: r = −0.06). In control subjects, no significant correlations were found between T2 relaxation times and either novelty seeking or harm avoidance in the mPFC, ACC or thalamus.

In chronic pain subjects NAA/Cr ratios within the mPFC were negatively correlated to novelty seeking (r = −0.55; Figure 5). That is, the greater the increase in NAA/Cr levels, the lower the individual’s novelty seeking score. Furthermore, there was a significant negative correlation between mPFC NAA/Cr and T2 relaxation times within the mPFC (r = −0.54). That is the greater the neural viability, the lower the T2 relaxation time. No significant relationship occurred between mPFC NAA/Cr levels and harm avoidance (r = 0.02). In control subjects, no significant relationship occurred between mPFC NAA/Cr and MI/Cr ratios and either novelty seeking (NAA/Cr: r = 0.19; MI/Cr: r = −0.12), or harm avoidance (NAA/Cr: r = −0.18; MI/Cr: r = −0.04).

## Discussion

Our results reveal that chronic pain is associated with significant anatomical changes within brain regions known to underpin an individual’s personality traits, i.e. the mPFC, ACC and mediodorsal thalamus. Strikingly, in chronic pain subjects only, the anatomical and biochemical changes within the mPFC and ACC were significantly correlated to the personality temperament of novelty seeking, with greater anatomical change associated with reduced novelty seeking. These data suggest that subtle change in brain anatomy may alter an individual’s personality characteristic.

Metz and colleagues [11] investigated the mPFC in the spared nerve injury model of neuropathic pain and reported that basal dendrites of pyramidal neurons in the contralateral mPFC, were longer, had more branches and had greater spine densities than those in sham-operated animals. Similar anatomical changes in humans would result in decreased mean diffusivity, since increased dendritic length and spine density would result in more restricted movement of free protons, and decreased T2 relaxation times, since axon and dendrite development results in increases in binding potential for protons of free water molecules to the surrounding macromolecules [13], [25], [26]. This is precisely what we found in our chronic pain subjects. Additionally, one would predict that such subtle changes in neuronal morphology would not result in a gross change in gray matter volume. Consistent with this we found that gray matter volume within the mPFC, as well as the ACC, and mediodorsal thalamus, did not display a significant change in chronic pain subjects compared with healthy pain-free controls.

Although taken alone, the mean diffusivity and T2 relaxation changes in our chronic pain subjects may reflect alterations in glia or blood vessels, our spectroscopy data reveals that these changes are also associated with increased NAA and no change in myoinositol. NAA occurs almost exclusively in neurons and is widely accepted as a marker of neuronal viability and synaptic health [27], [28]. Increased NAA levels are associated with better cognitive test performance and reduced levels occur in neurodegenerative diseases [29], [30]. In contrast, myoinositol is thought to be a glial marker, with elevated levels interpreted as gliosis in conditions such as Alzheimer’s disease [31], [32]. Although subject numbers of the spectroscopy scanning session were limited, we are confident that the changes in mean diffusivity and T2 relaxation reflect subtle neuronal changes such as altered basal dendritic length and spine density because we found increased levels of NAA and no change in myoinisotol within the mPFC.

It is becoming increasingly clear that chronic pain is characterized not only by an ongoing sensory and emotional experience, but also significant changes in an individual’s cognitive ability. For example, it has been reported that chronic pain subjects display impaired decision making in the Iowa Gambling Task, making disadvantageous choices that gained high immediate monetary returns at the risk of higher future losses, i.e., they were less sensitive to future consequences [4], [5]. Indeed, gross lesions of the mPFC and/or amygdala also result in decreased sensitivity to future consequences and poor performance on the Iowa Gambling Task [7], [8], [33]. Our chronic pain subjects had reduced novelty seeking and increased harm avoidance temperaments, a result consistent with a recent investigation, in which the personality profiles of over 200 chronic pain subjects were explored [6]. Furthermore, we found that novelty seeking, but not harm avoidance was significantly correlated to T2 relaxation times within the mPFC and ACC and NAA levels within the mPFC. Cloninger described novelty seeking as a “tendency toward intense exhilaration or excitement in response to novel stimuli … which leads to frequent exploratory activity in pursuit of potential rewards as well as active avoidance of monotony and potential punishment” [34]. The reduced novelty seeking in our chronic pain subjects implies that they are less likely to actively avoid monotony and potential punishment, which is consistent with being less sensitive to future consequences. Novelty seeking temperaments are thought to reflect the brain’s “behavioural activation” system [35] which depends on the integrity of the mesolimbic dopaminergic pathway, of which the mPFC and ACC are integral parts [36], [37]. In a number of psychiatric conditions, loss or impairment of PFC function is associated with cognitive deficits and conditions characterized by reduced dopaminergic activity such as Parkinson’s disease, are associated with reduced novelty seeking temperaments [38], [39].

The mPFC and ACC receive dopaminergic inputs directly from neurons within the ventral tegmental area. In experimental animals, ventral tegmental area lesions also result in cognitive deficits such as decreased exploration of environmental stimuli and reduced investigative behaviour [40]. Furthermore, dopamine depletion of the mPFC by 6-hydroxydopamine ventral tegmental area lesions, results in decreased basal dendritic lengths and spine densities of mPFC pyramidal cells [41]. Although in the opposite direction, these morphological changes are remarkably comparable in nature to those reported in the mPFC of experimental animals with neuropathic pain and supported by our MRI findings in humans with neuropathic pain. Importantly, given the unilateral nature of the mPFC anatomical changes that occur in the animal model of neuropathic pain (contralateral to injured nerve) and the unilateral nature of our MRI findings (contralateral to pain), it appears that the mPFC, ACC and thalamic anatomical changes associated with pain may result from the condition itself and may not pre-exist the injury/pain. It is possible that following neural injury, altered dopaminergic inputs result in increased basal dendrite lengths and increased spine densities of mPFC neurons, which in combination with inputs from other brain regions (e.g. amygdala), result in altered cognitive functions/temperaments such as novelty seeking and decision making ability. Interestingly, evidence is arising that chronic pain patients also have low Self-Directedness [6], [42]. Indeed, a low value in self-directedness can predict the presence of a personality disorder [20]. Future research should examine the interaction of Novelty Seeking and Self-Directedness on brain biochemistry, structure and function, particularly within the mPFC.

Our data reveal that subtle neural changes in the mPFC and ACC of individuals with chronic pain are associated with subtle differences in an individual’s personality profile. Of course it is well established that gross anatomical alterations in the prefrontal and cingulate cortices can drastically alter an individual’s personality; the case of Phineas Gage is a prime example. Although our data cannot implicate causality, it may provide an indication that even subtle brain alterations can change an individual’s personality. Interestingly, in healthy individuals the temperament Persistence has been shown to be associated with an overlapping circuit including the ACC, ventral striatum and the lateral orbital and mPFC [43], [44]. As this association is lacking in pain patients, this may show that the reduction in Novelty Seeking involving reduced active avoidance may be an adaptive process generated by subtle brain alterations after the development of neuropathic pain. Since the recent advent of more sensitive structural magnetic resonance imaging techniques, it is now known that many relatively common medical conditions are associated with significant changes in brain anatomy, including changes within the mPFC and ACC. For example, gray matter volumes in the mPFC and ACC are significantly altered in subjects with respiratory disorders [45], diabetes [46], osteoarthritis [47] and even obesity [48]. Given this, it is likely that most individuals will experience at least some subtle change in brain anatomy over their life time that may potentially result in a change in their personality profile. The idea that an individual’s personality is carried effectively unaltered throughout their adult life might need reconsideration.
